# Measurement of copy number variation in single cancer cells using rapid-emulsification digital droplet MDA

**DOI:** 10.1038/micronano.2017.18

**Published:** 2017-06-19

**Authors:** Samuel C. Kim, Gayatri Premasekharan, Iain C. Clark, Hawi B. Gemeda, Pamela L. Paris, Adam R. Abate

**Affiliations:** 1 Department of Bioengineering and Therapeutic Sciences, University of California, San Francisco (UCSF), California Institute for Quantitative Biosciences (QB3) San Francisco, San Francisco, CA 94158, USA; 2 Department of Urology, Division of Hematology & Oncology, University of California, San Francisco (UCSF), San Francisco, CA 94158, USA

**Keywords:** amplification bias, copy number variation, ddMDA, droplet microfluidics, multiple displacement amplification

## Abstract

Uniform amplification of low-input DNA is important for applications across biology, including single-cell genomics, forensic science, and microbial and viral sequencing. However, the requisite biochemical amplification methods are prone to bias, skewing sequence proportions and obscuring signals relating to copy number. Digital droplet multiple displacement amplification enables uniform amplification but requires expert knowledge of microfluidics to generate monodisperse emulsions. In addition, existing microfluidic methods are tedious and labor intensive for preparing many samples. Here, we introduce rapid-emulsification multiple displacement amplification, a method to generate monodisperse droplets with a hand-held syringe and hierarchical droplet splitter. Although conventional microfluidic devices require >10 min to emulsify a sample, our system requires tens of seconds and yields data of equivalent quality. We demonstrate the approach by using it to accurately measure copy number variation (CNV) in single cancer cells.

## Introduction

Sequencing is becoming an increasingly valuable tool in biology due to the universal importance of nucleic acids in living systems and the richness of the data it produces^1–3^. The system under investigation often contains tiny quantities of DNA, for example, in single-cell studies, and exponential amplification is required to obtain sufficient material for sequencing. However, exponential amplification reactions, such as polymerase chain reaction (PCR) or multiple displacement amplification (MDA), are prone to bias because molecules that begin amplifying sooner or have slightly higher doubling rates rapidly take over the system such that they comprise an inordinate proportion of the final population. Biased regions are sequenced at depth at the expense of other regions, producing uneven coverage that conceals important biological features, such as copy number variation (CNV).

An effective method to address this challenge and enable accurate and quantitative sequencing of single cells is to compartmentalize the reaction in millions of equally sized picoliter droplets, a process known as digital droplet MDA (ddMDA)^4–7^. In this approach, which is derived from the concept of digital MDA^8^, a sample of starting templates is emulsified through a microfluidic device such that each droplet contains a subset of the original template pool, typically one or a few molecules, with all the reagents necessary for MDA. The emulsion is then incubated, allowing the molecules to amplify. The compartmentalization eliminates competition between templates; molecules that start amplification early or amplify faster quickly reach saturation but do not take over the system, and molecules that amplify at slower rates can catch up. This scheme yields extremely uniform amplification and quantitative sequence data down to a single cell^4–6^.

A challenge with ddMDA is that it requires expert knowledge of microfluidics to emulsify the sample. Moreover, even with this knowledge, >10 min are required to emulsify 50 μL, making it tedious and time consuming to prepare many samples. Parallelized emulsifiers may be employed to address this problem^9–12^, but the devices are complicated to fabricate and require expert optimization and operation. A simple alternative is to compartmentalize the sample into polydisperse droplets generated by vortexing or pipetting. However, because the number of amplicons of a given template is proportional to the volume of the droplet containing it, volume polydispersity translates into amplification bias. Although this bias is substantially less than that of un-encapsulated MDA^6^, it nevertheless reduces the efficiency of the sequencing process and the quality of the sequencing data. To enable broader access to ddMDA and its powerful features, a new method is needed to easily and rapidly generate monodisperse droplets from DNA samples.

In this paper, we describe rapid-emulsification ddMDA (re-ddMDA), a method to generate monodisperse emulsions in a few seconds using a hand-operated microfluidic emulsifier. The sample to be emulsified is loaded into a syringe and injected by hand through the device, generating millions of monodisperse droplets in a few seconds. Because the droplets are monodisperse, amplification is uniform, yielding sequencing data comparable to painstakingly generated pump-driven emulsions. To demonstrate the efficacy of our approach, we apply it to measure CNV in single cancer cells and obtain results comparable to unamplified matched cancer genomes from millions of cells. Our method reduces the barrier to adopting ddMDA, enhances its scalability for preparing multiple samples, and should be valuable for implementation into high-throughput sequencing pipelines via interfacing with available liquid handling technologies such as pipetting robots.

## Materials and methods

### Device fabrication

The serial splitter device is fabricated using soft lithography. SU-8 3025 photoresist (MicroChem, Westborough, MA, USA) is used to make a 45-μm-tall master mold structure on a 3-inch silicon wafer using standard photolithography techniques. PDMS prepolymer (Momentive, Waterford, NY, USA; RTV 615) mixed with a curing agent at a 10:1 ratio is poured onto the master mold placed in a petri dish. After degassing under vacuum, the PDMS is cured at 65 °C for 1 h and removed by cutting. Holes are punched at inlet and outlet ports using a 0.75-mm biopsy punch (Ted Pella, Inc., Redding, CA, USA; Harris Uni-Core 0.75). After cleaning with scotch tape, the PDMS channel structure is bonded to a glass substrate by treating with oxygen plasma for 60 s at 1 mbar in a plasma cleaner (Harrick Plasma, Ithaca, NY, USA; PDC-001). The channel surface is treated with Aquapel to make it hydrophobic. For easy access to device fabrication, the CAD design file (Supplementary Figure S1) and a list of microfluidics foundries (Supplementary Table S1) are provided in Supplementary Information.

### VCaP cell culture

VCaP cell (ATCC, Manassas, VA, USA) is a prostate cancer cell line established from a vertebral metastatic lesion. The cells are maintained in Dulbecco's modified Eagle medium (DMEM) supplemented with 10% fetal bovine serum (FBS), 4 mM L-glutamine, 4500 mg L^−1^ glucose, 1 mM sodium pyruvate, 1500 mg L^−1^ sodium bicarbonate and 100 μg mL^−1^ streptomycin at 37 °C in a 5% CO_2_ incubator.

### FACS sorting of single cells

VCaP cells are released from culture with 0.25% trypsin, washed with PBS-0.2% BSA buffer and centrifuged at 1000 rpm for 5 min. The sample is re-suspended in ~300 μL PBS-0.2% BSA and analyzed by a FACS ARIA III (BD Biosciences, San Jose, CA, USA) equipped with 407-nm, 488-nm, 561-nm and 633-nm lasers. One, 10, and 50-cell aliquots are sorted at a slow speed under single-cell mode into 0.2-mL PCR tubes containing 5 μL of TE buffer placed in a 96-well plate holder.

### Array comparative genomic hybridization protocol

DNA from 5 million VCaP cells is extracted with the QIAamp DNA Blood Mini kit (Qiagen, Germantown, MD, USA). The final product is purified using the Qiagen PCR Purification Kit (Qiagen). DNA quality and quantity are assessed by UV-Vis spectrophotometry. Array comparative genomic hybridization (aCGH) is performed using a genome-wide oligonucleotide microarray platform (Human CGH 4×180 K microarray kit, Agilent Technologies, Santa Clara, CA, USA), following the manufacturer’s instructions. Human genomic DNA (G1471, Promega, Madison, WI, USA) is used as the control. Slides are scanned using an Agilent microarray scanner (model GC2505C), and images are processed using Feature Extraction CytoGenomics software (Agilent Technologies).

### re-ddMDA procedure

Before preparing reaction mixtures, all items that directly contact the reagents (syringes, tubings, and PCR tubes) are UV-treated for at least 30 min. FACS-sorted single cells are collected into 5 μL of TE buffer in a 0.2-mL PCR tube (Accuflow, E&K Scientific, Santa Clara, CA, USA). After adding 3 μL of D2 buffer (REPLI-g Single Cell, Qiagen), the tube is heated at 98 °C in a thermocycler for 4 min to lyse the cells and heat-fragment and denature gDNA. Then, 3 μL of STOP buffer (REPLI-g kit) is added to the tube to neutralize. Next, 40 μL of reaction mixture (29 μL reaction buffer, 9 μL water, 2 μL polymerase) is added to the tube on ice.

#### Pipette-push method

Briefly, 55 μL of fluorinated oil (HFE, 3 M Novec 7500, St Paul, MN, USA) supplemented with 2% (w/w) 008-FluoroSurfactant (RAN Biotechnologies, Beverly, MA, USA) is added to the MDA reaction mixture. The liquid is gently pipetted up and down 30 times using a 200-μL pipette (L-200XLS+, Mettler-Toledo Rainin, Oakland, CA, USA) set to 130 μL. Then, 60-inch long polyethylene tubing (SCI, Lake Havasu City, AZ, USA; PE/2, ID 0.38 mm, OD 1.09 mm) is attached to a 1-mL syringe (BD, Franklin Lakes, NJ, USA; 1-mL Luer-Lok syringe and 27-G 1/2 needle) with the plunger set to the 50 μL position. The pipetted MDA emulsion is withdrawn by moving the plunger to the 200 μL position while keeping the end of the tubing at the bottom of the PCR tube so that the oil is loaded first followed by the emulsion. Then, the reagent-loaded tubing is inserted into the inlet of the splitter device. The syringe plunger is push down to the 0 μL position to initiate flow, and the resulting split droplets are collected into a new PCR tube.

#### Suction-pull method

A flow-focus device (Supplementary Figure S1) is connected to the splitter device with PE/2 tubing in tandem. Gel-loading pipette tips are inserted into two inlet ports of the flow-focus device and serve as reservoirs. The MDA reaction mix and 110 μL of 2% (w/w) 008-FluoroSurfactant in HFE oil are added to the reservoirs. Seven-inch-long PE/2 tubing is attached to a 1-mL syringe, and the plunger is set to the 50 μL position. The tubing is inserted into the outlet of the splitter device and slowly pulled to the 200 μL position to initiate flow. As the emulsion fills the syringe, the plunger is pulled further to keep the suction pressure relatively constant. When all the MDA mix is injected, 20 μL of surfactant oil is added to the aqueous reservoir to continue oil flow and flush all remaining droplets into the collection syringe.

The prepared emulsion (in a PCR tube for the pipette-push method and in a syringe for suction-pull) is incubated at 30 °C for 16 h. Then, the enzyme is deactivated by heating at 70 °C for 20 min. The standard ddMDA samples are prepared as previously reported, and the re-ddMDA sample is prepared using the suction-pull method. The estimated numbers of template molecules per droplet are 0.11 and 1.2 for ddMDA (6 pL) and re-ddMDA (65 pL) droplets, respectively, assuming ~10 kb fragments of triploid VCaP genome.

### Library preparation and NGS

Droplets are coalesced by adding 100 μL perfluorooctanol (Sigma, 370533) and centrifuging at 1000 *g* for 1 min. The aqueous phase is transferred to a spin column for purification (Zymo Research, Irvine, CA, USA; DNA Clean and Concentrator). The purified DNA is quantified with fluorescence (Thermo Fisher Scientific, Waltham, MA, USA; Qubit dsDNA HS Assay Kit). DNA (1 ng) is tagmented following the manufacturer’s protocol (Illumina, San Diego, CA, USA; Nextera XT DNA Library Prep Kit) and purified with beads to select for ~300-bp fragments (AMPure XP, Beckman Coulter, Indianapolis, IN, USA). The library is characterized with a Bioanalyzer (Agilent, High Sensitivity DNA Analysis Kit) and quantified with qPCR (New England Biolabs, Ipswich, MA, USA; NEBNext Library Quant Kit for Illumina). Then, 15 pM library concentration is used for NGS runs on MiSeq sequencer (Illumina).

### Bioinformatics

The Fastq files are down-sampled using R (ShortRead package) to adjust total read counts for each sample to the same value (~2.8 million reads) and aligned to the human reference genome (UCSC hg19) using BWA Aligner (Illumina BaseSpace Labs, version 1.1.4). The coverage maps with 2.5 Mb window size for averaging are calculated from BAM files and visualized using R (GenomicAlignments and ggplot2 packages). The global mean coverage values for samples are ~0.1×. The Pearson correlation coefficients are calculated using cor() function of R’s stats package.

## Results and discussion

MDA is based on an enzymatic reaction catalyzed by φ29 DNA polymerase^13,14^. This highly processive polymerase has strand displacement activity, enabling isothermal amplification of input DNA with random hexamer primers. φ29 produces long amplicons (~10 kb) with low error rates, making MDA the method of choice for many low-input sequencing applications^15,16^. However, similar to most exponential reactions, MDA is prone to bias, skewing sequence proportions due to stochastic binding of the enzyme to the templates and preferential amplification of early-bound sequences^17^. To reduce bias, the amplification can be constrained by performing the reaction in microfluidic chambers^18,19^ or droplets^20^ that nevertheless yield sufficient DNA for sequencing. Alternatively, the sample can be divided and amplified in millions of monodisperse droplets, a method known as ddMDA, that produces superbly uniform sequencing data^4–7^.

**Figure 1:**
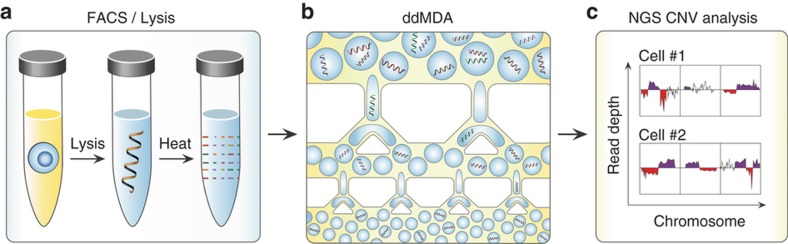
Rapid-emulsification ddMDA workflow. (**a**) Single cells are isolated by FACS into each well. Alkaline lysis at high temperature induces cell lysis, DNA denaturation, and fragmentation. (**b**) The sample is then rapidly emulsified through a microfluidic splitter and incubated for amplification. The amplified DNA is recovered from the droplets and sequenced (**c**).

To apply the ddMDA method to single cells, the first step is to isolate the cells in wells via fluorescence-activated cell sorting (FACS). Then, the cells are lysed, and their genomes are fragmented with high alkalinity and temperature (98 °C) for 4 min, cleaving genomic DNA (gDNA) into ~10-kb fragments (Figure 1a)^21,22^. The alkaline buffer is neutralized, and the MDA reagents are added. The sample is emulsified into millions of monodisperse droplets. With re-ddMDA, emulsification is accomplished by first generating a rough pipetted emulsion comprising large droplets and then monodispersely emulsifying it through a hierarchical splitting device by hand-injection with a syringe, requiring a few seconds (Figure 1b). The emulsion is incubated for 16 h at 30 °C to allow φ29 to amplify the single-molecule templates in the droplets. The droplets are chemically ruptured. The contents are pooled and subsequently processed for sequencing. When we start with a single-cell genome of ~6 picograms, ddMDA amplification results in greater than one microgram, providing ample DNA for library preparation, sequencing, and CNV measurements (Figure 1c).

**Figure 2:**
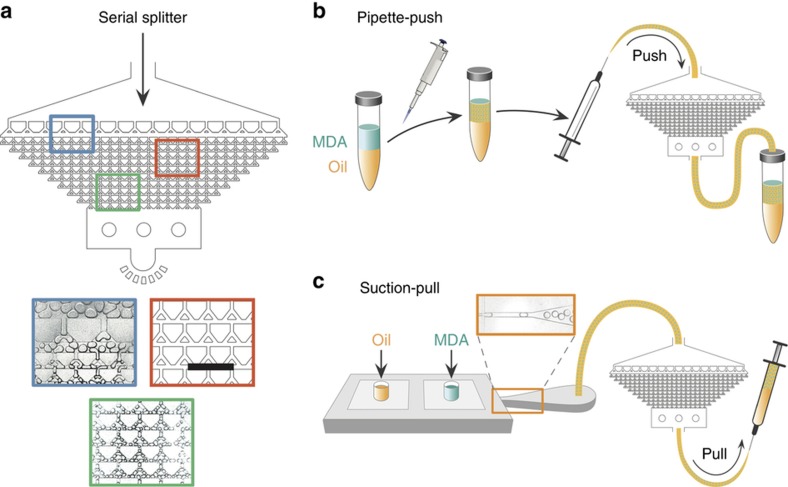
Methods for rapidly generating uniform emulsions for ddMDA. (**a**) A pre-formed emulsion comprising large, polydisperse droplets is introduced into the splitter device inlet and split to final droplets ~40 μm in diameter (**a**). The droplets can be generated either in positive pressure (**b**) or negative pressure (**c**) modes. In positive pressure mode, a coarse emulsion of the MDA sample is generated via pipetting, loaded into a syringe, and injected through the splitter. In negative pressure mode, the sample is loaded with oil into the inlets of a large droplet generator connected in series with the splitter, and the fluids are drawn through both devices by applying syringe suction. While the suction method generates more uniform emulsions, the positive pressure method is faster, emulsifying a sample of 50 μL in a few seconds. Scale bar in **a** is 600 μm.

Our rapid-emulsification device is based on geometrically mediated droplet breakup^23^, consisting of a sequence of channel bifurcations (Figure 2a). The device improves upon the premix emulsification method^24,25^, where pre-formed emulsions are dispersed further by flowing through a porous membrane. At a sufficiently high flow rate, the final droplet size asymptotes to the dimensions of the smallest channel in the hierarchy, such that initially large droplets split more times than small droplets, yielding a uniform emulsion. Thus, an advantage of this approach is that the final droplet size is insensitive to flow rate, allowing the injection to be performed by hand and obviating the need for microfluidic expertise or specialized pumps. The device also runs at high speeds (>10 000 μL h^−1^), generating millions of droplets in a few seconds. Combined, these features make rapid-emulsification ddMDA especially valuable for applications that require processing multiple samples.

We present two methods for rapidly preparing droplets for ddMDA. In the pipette-push method (Figure 2b), pipetting by hand generates large polydisperse droplets with a broad size distribution. The polydisperse emulsion is then processed through the splitter, generating a uniform emulsion with rare instances of large droplets. Injecting a pipetted emulsion through the splitter is easy and fast and yields reasonably uniform droplets for ddMDA. However, the starting pipetted emulsion may vary between users. Moreover, occasional very large droplets are not completely fragmented to the final size, resulting in some polydispersity. Hence, as an alternative approach, we also emulsify the sample using a tandem device consisting of a droplet generator and a splitter operated by a hand-held syringe (Figure 2c). The ddMDA reagents are loaded into the inlets of the droplet generator connected to the splitter through a tube. A syringe is connected to the splitter outlet to generate a vacuum, providing suction that draws the fluids through the droplet generator and splitter. Because the droplet generator forms monodisperse large droplets (Figure 2c, orange panel) and the splitter operates at reasonably constant flow rates, these emulsions are even more uniform than the emulsions formed by the pipette-push method. However, in return for these benefits, the suction-pull method is slower, requiring tens of seconds to emulsify a 50-μl sample.

**Figure 3:**
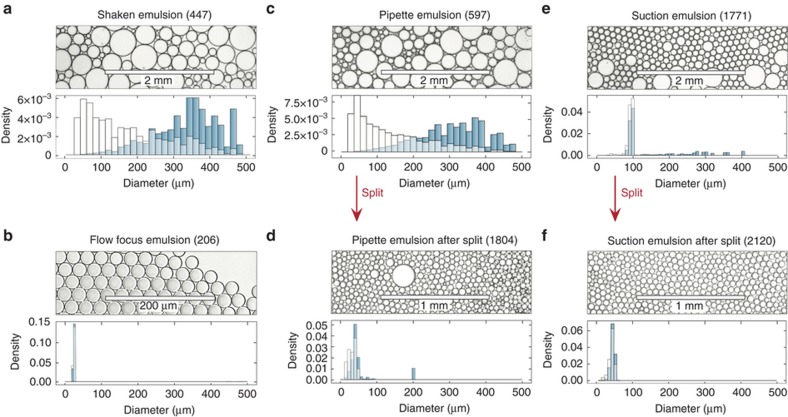
Assessment of droplet quality prepared by different methods. Shaken emulsions are easy and fast to make but polydisperse, resulting in bias (**a**). Microfluidic flow focusing, by contrast, requires specialized pumps and is relatively slow (>10 min for a 50 μL sample) but provides exceedingly uniform droplets and superbly uniform sequencing data. (**b**) Hand-injection of a polydisperse emulsion generated by pipetting (**c**) yields reasonably uniform droplets (**d**) with greater polydispersity than flow focusing but much less than shaking. Negative pressure generation in a tandem flow focus (**e**) and splitter (**f**) yields even more uniform emulsions, although this sacrifices some speed. The volume-weighted histogram (blue bars) illustrates expected amplification bias resulting from polydispersity, since product numbers scale with encapsulating droplet volume. Numbers in parentheses are counts of analyzed droplets.

To assess the effectiveness of these methods for generating emulsions, we compare size distributions of the resultant droplets (Figure 3). The simplest and fastest method to generate an emulsion for ddMDA is to vortex the ddMDA mix with oil and surfactant. However, the resultant droplets are extremely polydisperse, as shown in Figure 3a, and consequently result in bias^6^. In contrast, a flow-focus microfluidic droplet generator operated at controlled flow rates using syringe pumps can form exquisitely monodisperse emulsions (Figure 3b) that yield ideal data^4–6^; however, the requirement of pumps and slow rate of formation limit this method’s broad adoption. Indeed, the sequencing process itself introduces bias from multiple sources, including the MDA reaction’s preference for amplifying certain sequences, limited cycle PCR during library preparation, and systematic errors produced by the sequencer itself^26^. Consequently, such extreme monodispersity may not be necessary to produce the best possible data. Rather, the optimal method is one that obtains these data in the most convenient and fastest protocol possible.

Rapid-emulsification ddMDA accomplishes this by trading impeccable monodispersity for a simplified workflow and markedly faster emulsification. The sample is first coarsely emulsified by hand using a pipette, generating large, polydisperse droplets with a broad size distribution (Figure 3c). The polydisperse emulsion is then processed through the splitter, generating a uniform emulsion with rare instances of large droplets (Figure 3d). The remaining polydispersity can be traced to extremely large droplets in the pipetted emulsion with diameters >350 μm. Our splitter contains 11 sequential splits, yielding a 2^11^-fold reduction in volume and ~13-fold reduction in diameter. Hence, droplets larger than 350 μm do not reduce to the final ~30 μm size, resulting in some large droplets. Nevertheless, rare droplets are massively outnumbered by correctly sized droplets and thus do not significantly affect data quality. When the suction-pull method is used, the droplets processed through the splitter almost never exceed the maximum size (Figure 3e), resulting in an even more uniform final emulsion (Figure 3f).

An important area in which accurate and quantitative sequencing of low-input DNA is necessary is single cancer cell genomics. Solid tumors shed cells into a patient’s blood stream, called circulating tumor cells (CTCs). Many technologies are available for enriching CTCs and can recover cells from patients with metastases for many different types of cancers^27–29^. Moreover, because CTCs originate from a tumor, they may share similar genetic and phenotypic characteristics, affording the potential to obtain detailed information about the tumor without the need to procure tissue biopsies that are rarely performed due to difficulty, cost and morbidity^30^. A particularly important genomic feature of many cancers is CNV, in which certain regions of the genome are duplicated or deleted. CNV is important because edits of the genome that change the counts of sequences are thought to more likely yield selectable phenotypes than mutations that alter gene sequences^31^. In addition, CNV correlates with the metastatic and evolutionary potential of numerous cancers, making it a potentially valuable biomarker for cancer diagnostics^32^. However, measuring CNV is challenging because the single-cell genome must be massively amplified to yield sufficient DNA for sequencing, often destroying the valuable CNV information. Here, ddMDA’s ability to uniformly amplify minute quantities of DNA enables accurate CNV measurements of single cancer cells^4^.

**Figure 4:**
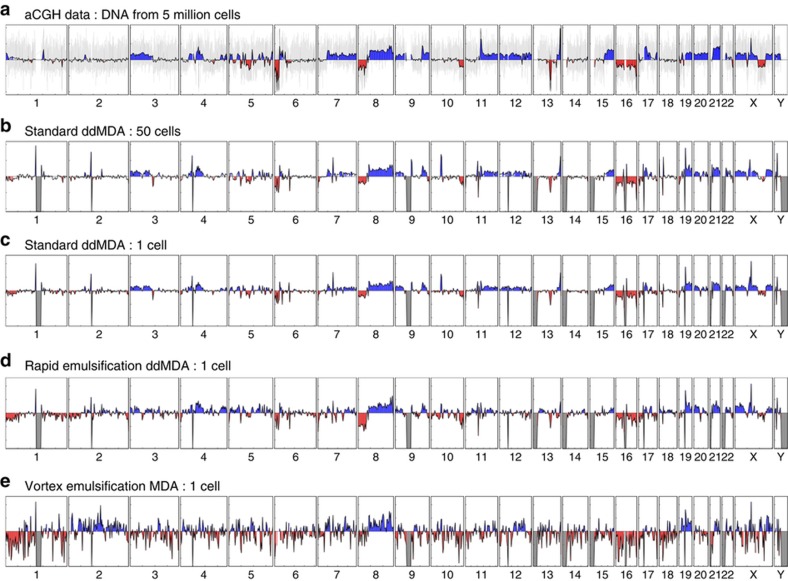
Rapid-emulsification ddMDA produces CNV measurements comparable to microfluidic ddMDA and rivaling measurements based on unamplified DNA. (**a**) An aCGH microarray is used to measure CNV in gDNA extracted from five million VCaP cells (2.5 Mb averaging window); the *y* axis is log scale, indicating the signal difference between VCaP gDNA and the control gDNA from a normal cell line, and the gray background shows the raw signals from individual probes before averaging. (**b**) Similar maps can be generated from low-coverage sequencing data for 50 cells subjected to ddMDA with microfluidic flow focusing emulsions. (**c**) The low bias of ddMDA even allows accurate CNV measurements from a single cancer cell. (**d**) Suction-generated emulsions are also uniform and, thus, produce data of equivalent quality. (**e**) Emulsions prepared by vortexing produce data with a large degree of noise due to amplification bias. **c**–**e** show average data from two independent measurements of single cells. The global mean coverage values for samples in **b**–**d** are 0.098×, 0.089×, and 0.099×, respectively.

To test whether re-ddMDA enables single-cell CNV measurements, we apply it to cancer cells from the VCaP cell line. As a control, we collect total DNA from five million VCaP cells and perform aCGH, the gold standard in characterizing CNV for cultivable cancer cells (Figure 4a). The aCGH array provides CNV measurements with a theoretical resolution of ~100 kb estimated from the median distance of 13 kb between each hybridization probe on the 4×180 K array.

To confirm that similar measurements can be obtained from sequencing data, we apply standard microfluidic ddMDA with monodisperse 26-μm droplets to gDNA from 50 VCaP cells. Sequence amplifications (blue), deletions (red) and long-range dropouts (gray) are marked for the aCGH and ddMDA data. As expected, we observe excellent correspondence between the ddMDA sequence data and aCGH reference (Figure 4b), illustrating the power of ddMDA with uniform droplets. A powerful feature of ddMDA is that it allows accurate sequencing of single cells. To confirm this feature, we repeat the measurement on a single VCaP cell isolated by FACS, again marking copy number signatures and observing excellent correspondence with the 50 and 5 million cell data (Figure 4c). To determine whether our re-ddMDA approach provides the uniformity necessary to obtain accurate CNV measurements, we also apply it to a single VCaP cell (Figure 4d). Again, we find excellent agreement with the control samples, illustrating re-ddMDA’s effectiveness for measuring CNVs in single cells. As expected, the data from vortexed emulsions exhibit larger variation and noise in the read depth profile, resulting in poorer CNV detection (Figure 4e). We also compute the Pearson correlation coefficients against the aCGH data to quantitatively assess the similarities between each measurement (Supplementary Tables S2, S3, and S4). Although the 50-cell ddMDA data show the highest correlation with the aCGH data (*r*=0.67), the single-cell ddMDA data (*r*=0.66) and the single-cell re-ddMDA data (*r*=0.52) yield similar correlation coefficients, confirming the consistency of copy number information between the methods. The vortexed emulsion data yields the lowest value (*r*=0.31). The correlation between ddMDA and re-ddMDA is higher, as indicated by the correlation coefficients against the 50-cell ddMDA data: 0.96 and 0.82 for 1-cell ddMDA and 1-cell re-ddMDA, respectively. The sequencing data are obtained at very-low coverage (~0.1×) and are not processed further for bias correction or normalization. The correspondence between aCGH and the ddMDA methods may improve with greater sequencing coverage or by employing more sophisticated CNV detection algorithms, such as GC bias correction and segmentation with variable bin size^33,34^.

## Conclusions

Uniform amplification of low-input DNA is important for a variety of applications, including hybridization array analysis and next-generation sequencing. Existing ddMDA methods require microfluidic expertise and are limited in speed. Here, we demonstrate that emulsification of samples with hand-operated syringes and a simple microfluidic droplet splitter can generate emulsions that yield data of similar quality. In addition to being simpler to adopt, our approach can emulsify samples in a few seconds, making it valuable for preparing multiple samples. Although our approach requires access to a microfluidic device consisting of a bifurcating channel network, the device is simple to fabricate and could easily be constructed and purchased from existing commercial vendors. Although we use hand-held syringes to operate the device, it should also be possible to do so using a pipette by integrating the device into a disposable pipette tip.

Our data on a metastatic cancer cell line provide initial support for application of this approach to conducting CNV measurements of single cells. Further studies using CTCs from patient samples will provide valuable genomic information for medical treatment. Our method should be useful for applications requiring uniform sequence data from minimal starting material and those applications in which speed and convenience are important factors.

## References

[bib1] Shapiro E, Biezuner T, Linnarsson S. Single-cell sequencing-based technologies will revolutionize whole-organism science. Nature Reviews Genetics 2013; 14: 618–630. 10.1038/nrg3542 23897237

[bib2] van Dijk EL, Auger H, Jaszczyszyn Y et al. Ten years of next-generation sequencing technology. Trends in Genetics 2014; 30: 418–426. 25108476 10.1016/j.tig.2014.07.001

[bib3] Shendure J, Ji H. Next-generation DNA sequencing. Nature Biotechnology 2008; 26: 1135–1145. 10.1038/nbt1486 18846087

[bib4] Fu Y, Li C, Lu S et al. Uniform and accurate single-cell sequencing based on emulsion whole-genome amplification. Proceedings of the National Academy of Sciences of the United States of America 2015; 112: 11923–11928. 26340991 10.1073/pnas.1513988112 PMC4586872

[bib5] Nishikawa Y, Hosokawa M, Maruyama T et al. Monodisperse picoliter droplets for low-bias and contamination-free reactions in single-cell whole genome amplification. PLoS ONE 2015; 10: e0138733. 26389587 10.1371/journal.pone.0138733 PMC4577099

[bib6] Sidore AM, Lan F, Lim SW et al. Enhanced sequencing coverage with digital droplet multiple displacement amplification. Nucleic Acids Research 2016; 44: e66. 26704978 10.1093/nar/gkv1493 PMC4838355

[bib7] Rhee M, Light YK, Meagher RJ et al. Digital droplet multiple displacement amplification (ddMDA) for whole genome sequencing of limited DNA samples. PLoS ONE 2016; 11: e0153699. 27144304 10.1371/journal.pone.0153699 PMC4856258

[bib8] Blainey PC, Quake SR. Digital MDA for enumeration of total nucleic acid contamination. Nucleic Acids Research 2011; 39: e19. 21071419 10.1093/nar/gkq1074 PMC3045575

[bib9] Romanowsky MB, Abate AR, Rotem A et al. High throughput production of single core double emulsions in a parallelized microfluidic device. Lab on a Chip 2012; 12: 802–807. 22222423 10.1039/c2lc21033a

[bib10] Lim J, Caen O, Vrignon J et al. Parallelized ultra-high throughput microfluidic emulsifier for multiplex kinetic assays. Biomicrofluidics 2015; 9: 034101. 26015838 10.1063/1.4919415 PMC4425725

[bib11] Conchouso D, Castro D, Khan SA et al. Three-dimensional parallelization of microfluidic droplet generators for a litre per hour volume production of single emulsions. Lab on a Chip 2014; 14: 3011–3020. 24947654 10.1039/c4lc00379a

[bib12] Nisisako T, Torii T. Microfluidic large-scale integration on a chip for mass production of monodisperse droplets and particles. Lab on a Chip 2008; 8: 287–293. 18231668 10.1039/b713141k

[bib13] Blanco L, Bernad A, Lázaro JM et al. Highly efficient DNA synthesis by the phage phi 29 DNA polymerase. Symmetrical mode of DNA replication. Journal of Biological Chemistry 1989; 264: 8935–8940. 2498321

[bib14] Dean FB, Nelson JR, Giesler TL et al. Rapid amplification of plasmid and phage DNA using Phi29 DNA polymerase and multiply-primed rolling circle amplification. Genome Research 2001; 11: 1095–1099. 11381035 10.1101/gr.180501 PMC311129

[bib15] Binga EK, Lasken RS, Neufeld JD. Something from (almost) nothing: The impact of multiple displacement amplification on microbial ecology. The ISME Journal 2008; 2: 233–241. 18256705 10.1038/ismej.2008.10

[bib16] Lasken RS. Genomic sequencing of uncultured microorganisms from single cells. Nature Reviews Microbiology 2012; 10: 631–640. 22890147 10.1038/nrmicro2857

[bib17] Raghunathan A, Ferguson HR, Bornarth CJ et al. Genomic DNA amplification from a single bacterium. Applied and Environmental Microbiology 2005; 71: 3342–3347. 15933038 10.1128/AEM.71.6.3342-3347.2005 PMC1151817

[bib18] Marcy Y, Ishoey T, Lasken RS et al. Nanoliter reactors improve multiple displacement amplification of genomes from single cells. PLoS Genetics 2007; 3: e155. 10.1371/journal.pgen.0030155 PMC1988849 17892324

[bib19] Marcy Y, Ouverney C, Bik EM et al. Dissecting biological ‘dark matter’ with single-cell genetic analysis of rare and uncultivated TM7 microbes from the human mouth. Proceedings of the National Academy of Sciences of the United States of America 2007; 104: 11889–11894. 17620602 10.1073/pnas.0704662104 PMC1924555

[bib20] Leung K, Klaus A, Lin BK et al. Robust high-performance nanoliter-volume single-cell multiple displacement amplification on planar substrates. Proceedings of the National Academy of Sciences of the United States of America 2016; 113: 201520964. 10.1073/pnas.1520964113 PMC4968760 27412862

[bib21] Yang Y, Hang J. Fragmentation of genomic DNA using microwave irradiation. Journal of Biomolecular Techniques 2013; 24: 98–103. 23814501 10.7171/jbt.13-2402-005 PMC3671502

[bib22] Liu P, Li X, Greenspoon SA et al. Integrated DNA purification, PCR, sample cleanup, and capillary electrophoresis microchip for forensic human identification. Lab on a Chip 2011; 11: 1041. 21293830 10.1039/c0lc00533a

[bib23] Link DR, Anna SL, Weitz DA et al. Geometrically mediated breakup of drops in microfluidic devices. Physical Review Letters 2004; 92: 054503. 14995311 10.1103/PhysRevLett.92.054503

[bib24] Nazir A, Boom RM, Schroën K. Droplet break-up mechanism in premix emulsification using packed beds. Chemical Engineering Science 2013; 92: 190–197.

[bib25] Hornig N, Fritsching U. Liquid dispersion in premix emulsification within porous membrane structures. Journal of Membrane Science 2016; 514: 574–585.

[bib26] Aird D, Ross MG, Chen W-S et al. Analyzing and minimizing PCR amplification bias in Illumina sequencing libraries. Genome Biology 2011; 12: R18. 21338519 10.1186/gb-2011-12-2-r18 PMC3188800

[bib27] Friedlander TW, Premasekharan G, Paris PL. Looking back, to the future of circulating tumor cells. Pharmacology & Therapeutics 2014; 142: 271–280. 24362084 10.1016/j.pharmthera.2013.12.011

[bib28] Yu M, Stott S, Toner M et al. Circulating tumor cells: Approaches to isolation and characterization. The Journal of Cell Biology 2011; 192: 373–382. 21300848 10.1083/jcb.201010021 PMC3101098

[bib29] Alunni-Fabbroni M, Sandri MT. Circulating tumour cells in clinical practice: Methods of detection and possible characterization. Methods 2010; 50: 289–297. 20116432 10.1016/j.ymeth.2010.01.027

[bib30] Maheswaran S, Haber DA. Circulating tumor cells: A window into cancer biology and metastasis. Current Opinion in Genetics & Development 2010; 20: 96–99. 20071161 10.1016/j.gde.2009.12.002 PMC2846729

[bib31] Shlien A, Malkin D. Copy number variations and cancer. Genome Medicine 2009; 1: 62. 19566914 10.1186/gm62 PMC2703871

[bib32] Zack TI, Schumacher SE, Carter SL et al. Pan-cancer patterns of somatic copy number alteration. Nature Genetics 2013; 45: 1134–1140. 24071852 10.1038/ng.2760 PMC3966983

[bib33] Abyzov A, Urban AE, Snyder M et al. CNVnator: An approach to discover, genotype, and characterize typical and atypical CNVs from family and population genome sequencing. Genome Research 2011; 21: 974–984. 21324876 10.1101/gr.114876.110 PMC3106330

[bib34] Garvin T, Aboukhalil R, Kendall J et al. Interactive analysis and assessment of single-cell copy-number variations. Nature Methods 2015; 12: 1058–1060. 26344043 10.1038/nmeth.3578 PMC4775251

