# Supplementary material for: Measurement of copy number variation in single cancer cells using rapid-emulsification digital droplet MDA
Source: Microsyst Nanoeng. 2017 Jun 19;3:17018. doi: 10.1038/micronano.2017.18 (PMC6108428; doi:10.1038/micronano.2017.18)
Supplement: Supplementary Information [file micronano201718-s2.pdf]

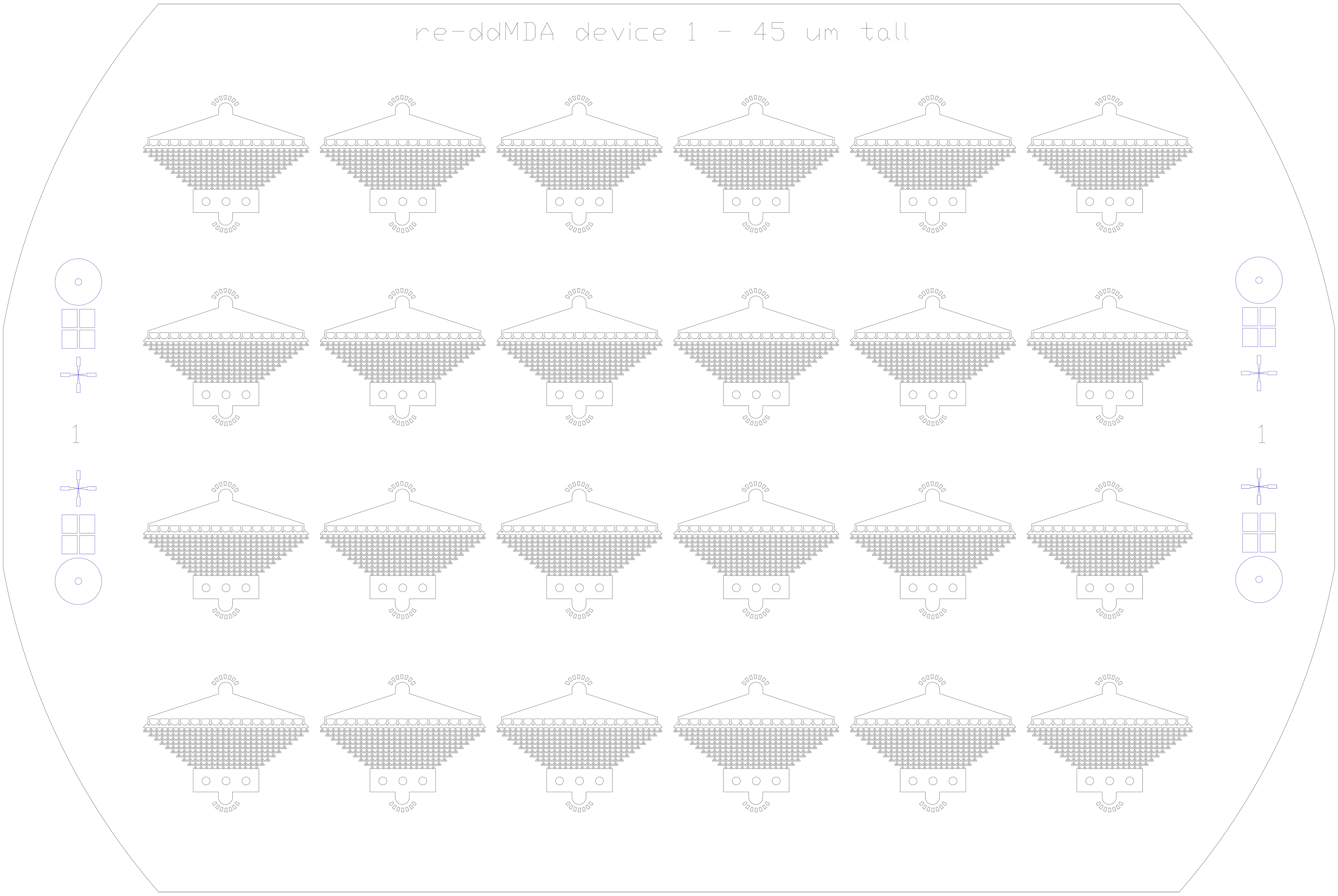
re-ddMDA device 1 - 45 um tall
1
1

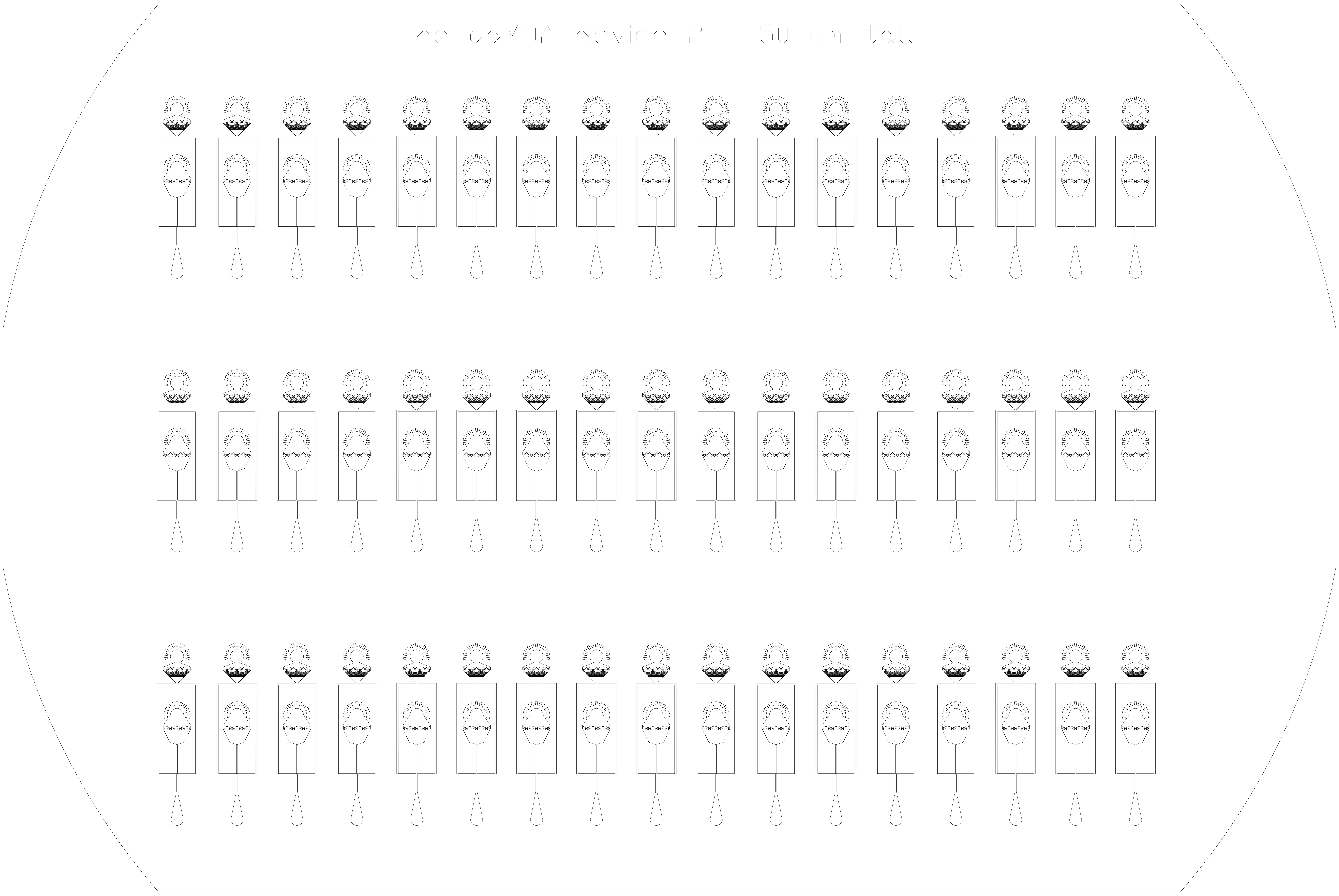
re-ddMDA device 2 - 50 um tall
