# Supplementary material for: Measurement of copy number variation in single cancer cells using rapid-emulsification digital droplet MDA
Source: Microsyst Nanoeng. 2017 Jun 19;3:17018. doi: 10.1038/micronano.2017.18 (PMC6108428; doi:10.1038/micronano.2017.18)
Supplement: Supplementary Figure and Tables [file micronano201718-s1.pdf]

Supplementary file

Measurement of copy number variation in single cancer cells using rapid-emulsification digital droplet MDA

Samuel C. Kim[1,*], Gayatri Premasekharan[2,*], Iain C. Clark[1], Hawi B. Gemeda[1], Pamela L. Paris[2] and Adam R. Abate[1]

Microsystems & Nanoengineering (2017) **3,** 17018; doi:10.1038/micronano.2017.18; Published online: 19 June 2017

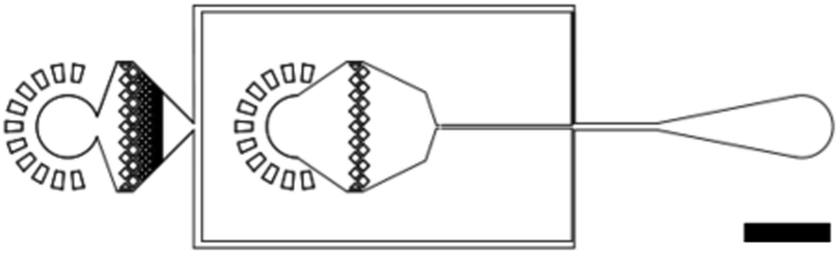

Figure S1 Flow focus device used for suction-pull method (scale bar: 1 mm). Nozzle size is 50 μm (*H*) × 80 μm (*W*). The CAD file is also provided as Supplementary Data (re-ddMDA_device.dwg).

Table S1 Academic and commercial microfluidics foundries

Name	Web
Achira Labs (India)	http://www.achiralabs.com/microfluidic-chip-fabrication/
CiDRA Precision Services (USA)	http://www.cidraprecisionservices.com/
Elveflow (France)	http://www.elveflow.com/
FlowJEM (Canada)	http://www.flowjem.com/
Kavli Nanoscience Institute (USA)	http://www.kni.caltech.edu/facilities/
SIMTech Microfluidics Foundry (Singapore)	http://www1.simtech.a-star.edu.sg/smf/
uFluidix (USA)	http://ufluidix.com/

Table S2 Correlation coefficients between CNV data obtained from different methods

	aCGH	ddMDA-50cell	ddMDA-1cell	re-ddMDA-1cell	vortex-MDA-1cell
aCGH	1	0.669	0.662	0.524	0.314
ddMDA-50cell		1	0.959	0.822	0.467
ddMDA-1cell			1	0.829	0.478
re-ddMDA-1cell				1	0.432

2.5 Mb binned log ratios were used to compute correlation coefficient.

Table S3 Correlation coefficients between CNV data obtained from different methods

	aCGH	ddMDA-50cell	ddMDA-1cell	re-ddMDA-1cell	vortex-MDA-1cell
aCGH	1	0.635	0.609	0.450	0.167
ddMDA-50cell		1	0.877	0.693	0.202
ddMDA-1cell			1	0.674	0.190
re-ddMDA-1cell				1	0.154

500 kb binned log ratios were used to compute correlation coefficient.

[1]Department of Bioengineering and Therapeutic Sciences, University of California, San Francisco (UCSF), California Institute for Quantitative Biosciences (QB3) San Francisco, San Francisco, CA 94158, USA and [2]Department of Urology, Division of Hematology & Oncology, University of California, San Francisco (UCSF), San Francisco, CA 94158, USA. Correspondence: Adam R. Abate (adam@abatelab.org)
*These authors contributed equally to this work.

Table S4 Correlation coefficients between CNV data obtained from different methods

	aCGH	ddMDA-50cell	ddMDA-1cell	re-ddMDA-1cell	vortex-MDA-1cell
aCGH	1	0.557	0.460	0.327	0.127
ddMDA-50cell		1	0.609	0.476	0.110
ddMDA-1cell			1	0.398	0.077
re-ddMDA-1cell				1	0.076

100 kb binned log ratios were used to compute correlation coefficient.
